# Supplementary material for: Expression of Ceramide-Metabolizing Enzymes in the Heart Adipose Tissue of Cardiovascular Disease Patients
Source: Int J Mol Sci. 2023 May 30;24(11):9494. doi: 10.3390/ijms24119494 (PMC10254038; doi:10.3390/ijms24119494)

Supplementary Figure S1. Representative example of total protein normalization. Uncropped Western blot and total protein normalization image for the quantification of SPTLC1 expression in subcutaneous adipose tissue (SAT), epicardial adipose tissue (EAT), and perivascular adipose tissue (PVAT) in patients with coronary artery disease (CAD)

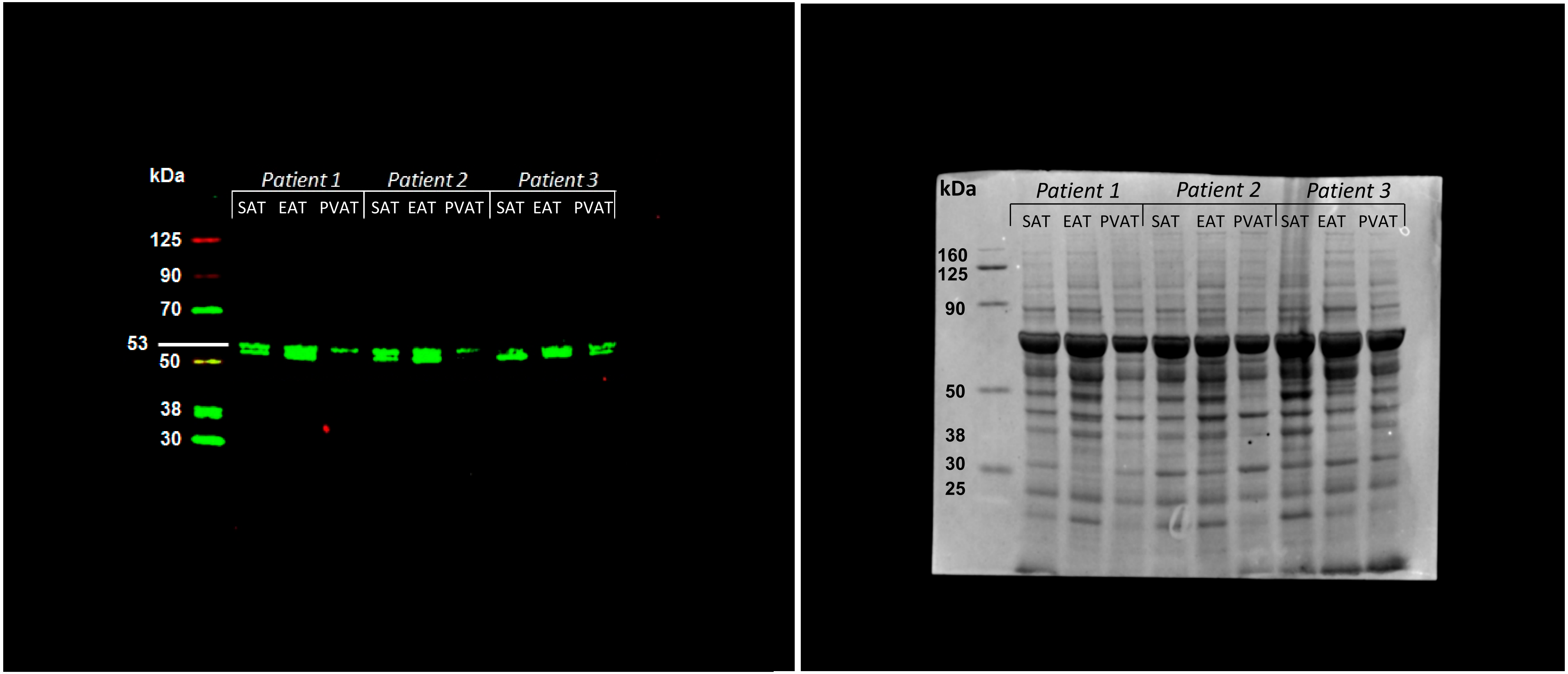

Supplementary Figure S2. Representative example of total protein normalization. Uncropped Western blot and total protein normalization image for the quantification of SPTLC1 expression in subcutaneous adipose tissue (SAT), epicardial adipose tissue (EAT), and perivascular adipose tissue (PVAT) in patients with valvular heart disease (VHD)

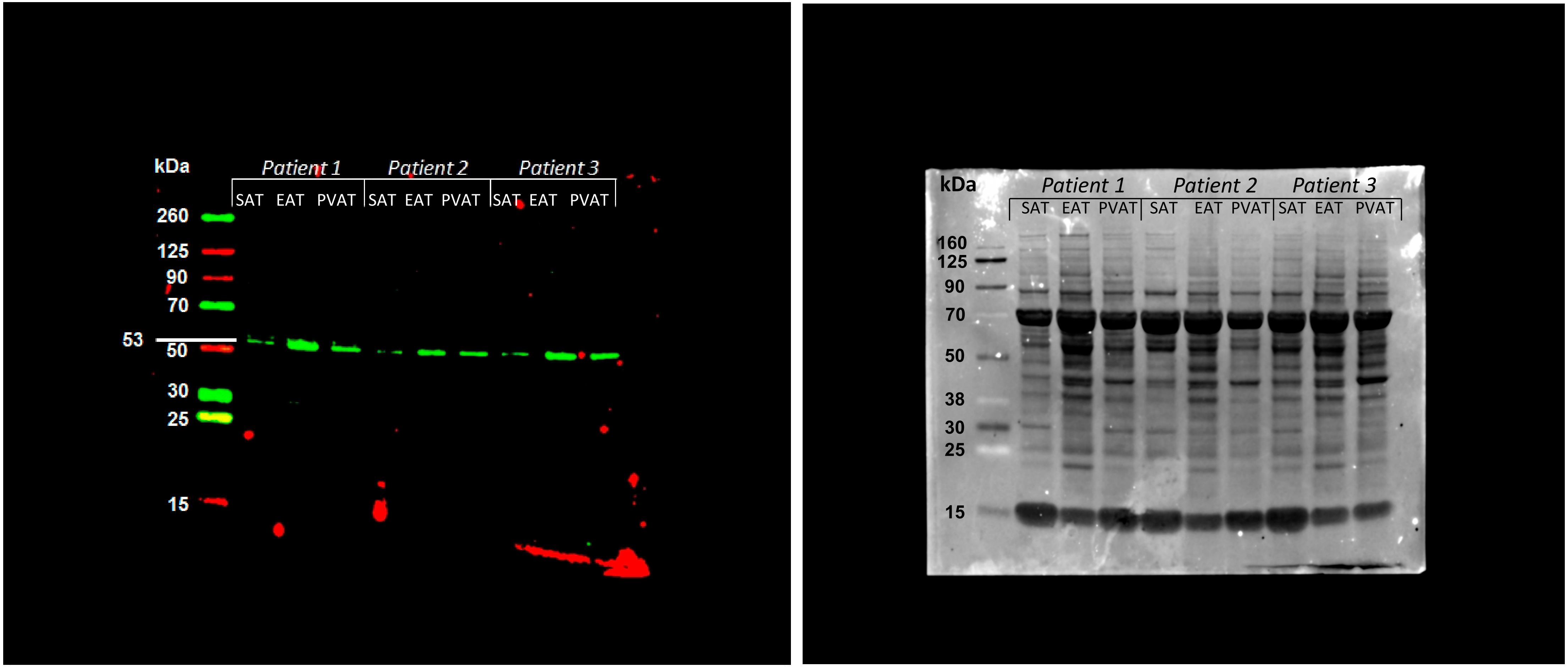

Supplementary Figure S3. Uncropped Western blot of SPTLC2 expression in subcutaneous adipose tissue (SAT), epicardial adipose tissue (EAT), and perivascular adipose tissue (PVAT) in patients with coronary artery disease (CAD)

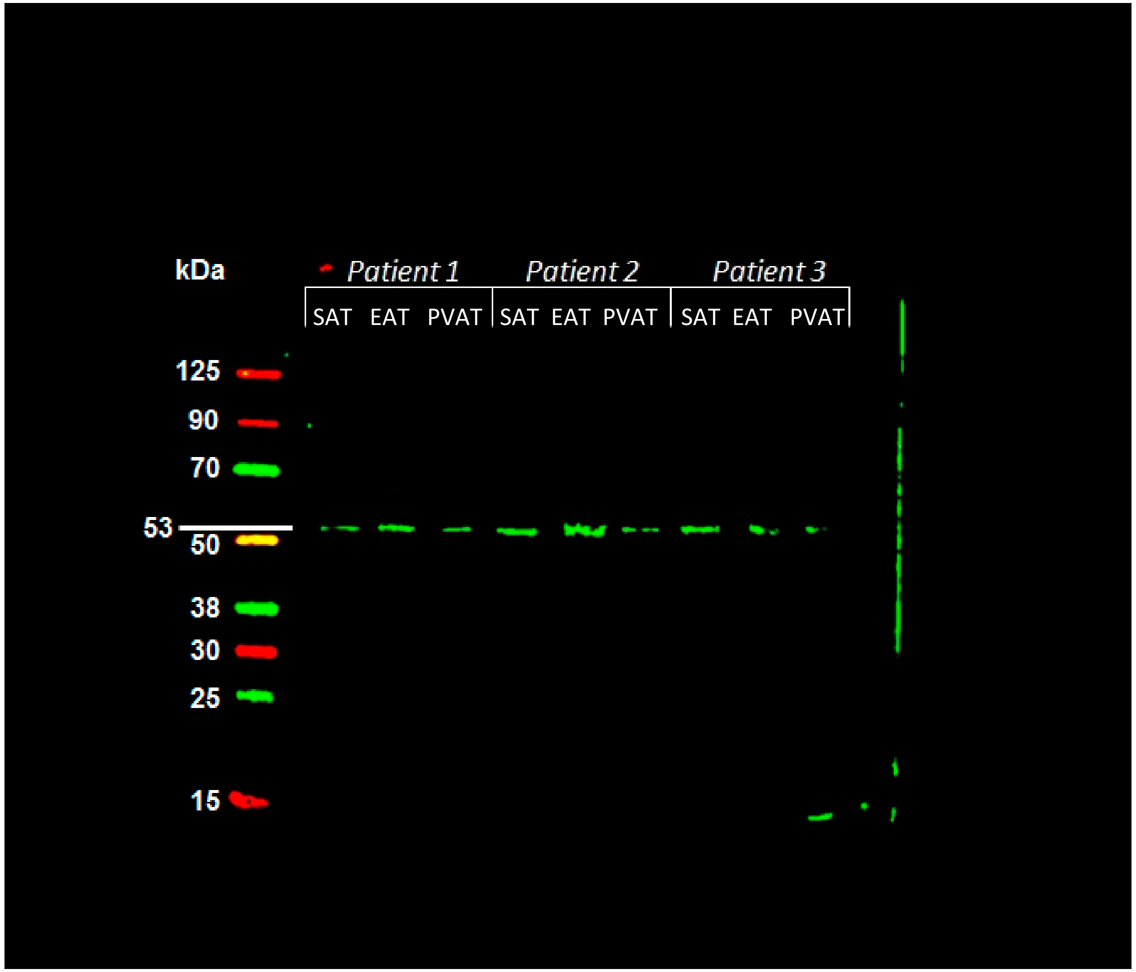

Supplementary Figure S4. Uncropped Western blot of SPTLC2 expression in subcutaneous adipose tissue (SAT), epicardial adipose tissue (EAT), and perivascular adipose tissue (PVAT) in patients with valvular heart disease (VHD)

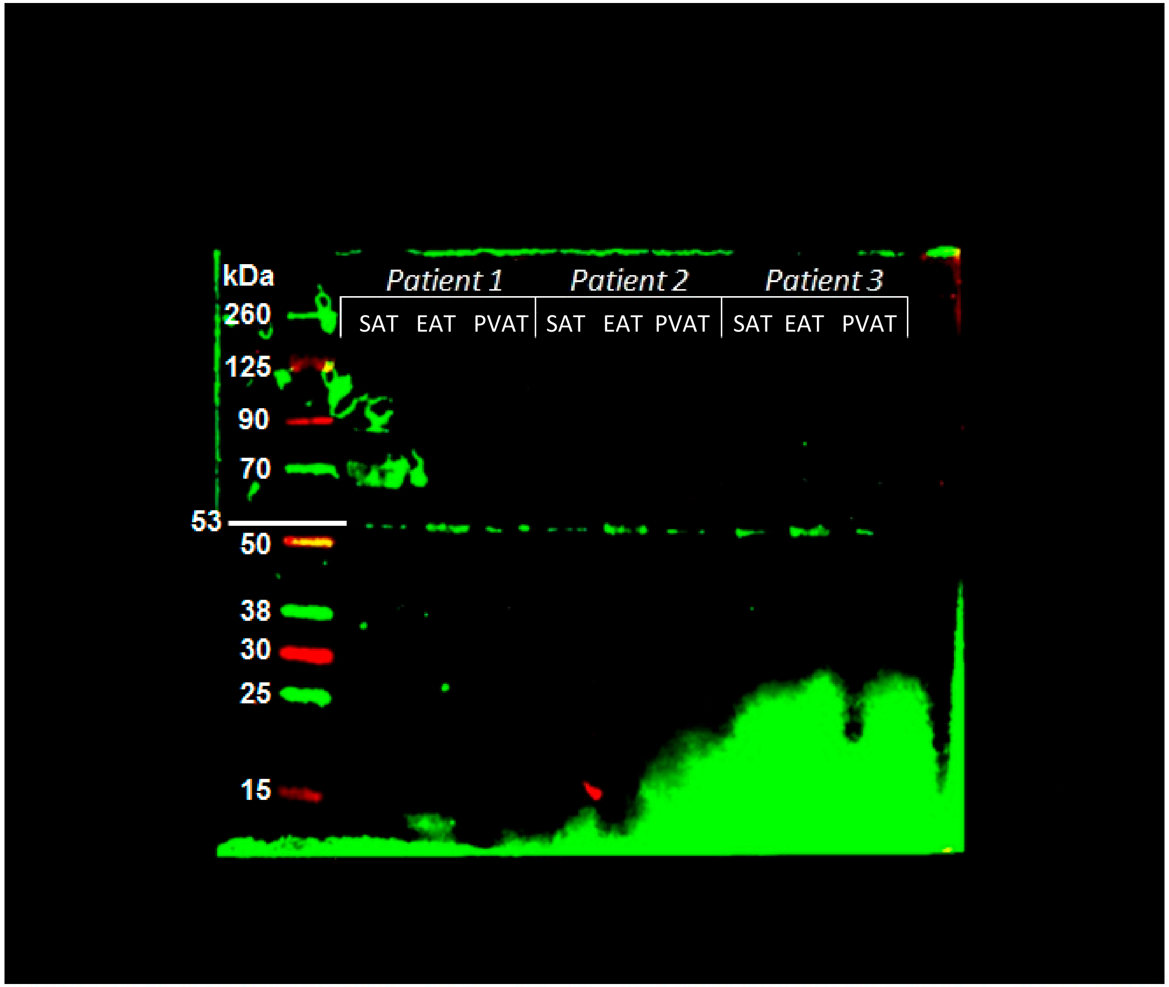

Supplementary Figure S5. Uncropped Western blot of CERS6 expression in subcutaneous adipose tissue (SAT), epicardial adipose tissue (EAT), and perivascular adipose tissue (PVAT) in patients with coronary artery disease (CAD)

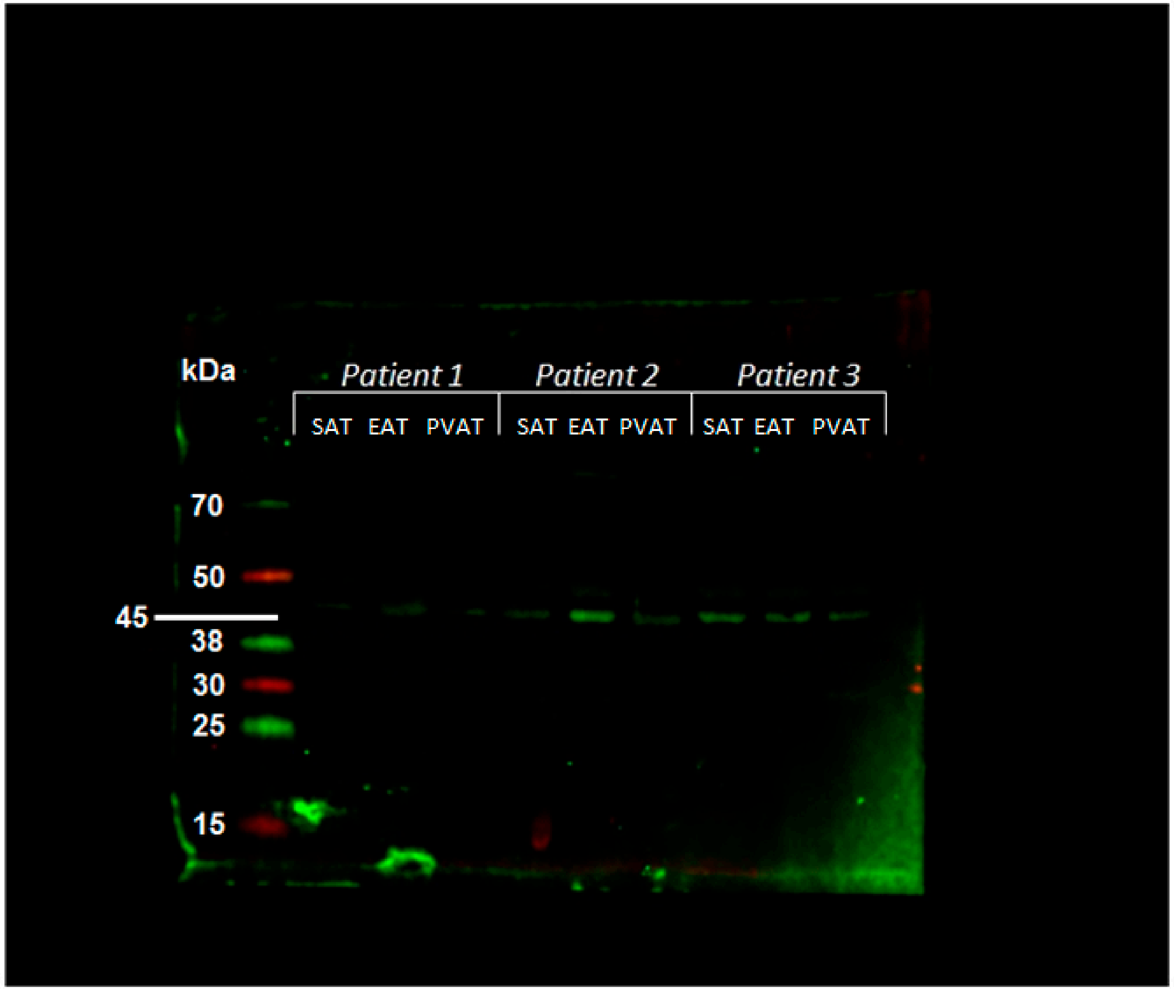

Supplementary Figure S6. Uncropped Western blot of CERS6 expression in subcutaneous adipose tissue (SAT), epicardial adipose tissue (EAT), and perivascular adipose tissue (PVAT) in patients with valvular heart disease (VHD)

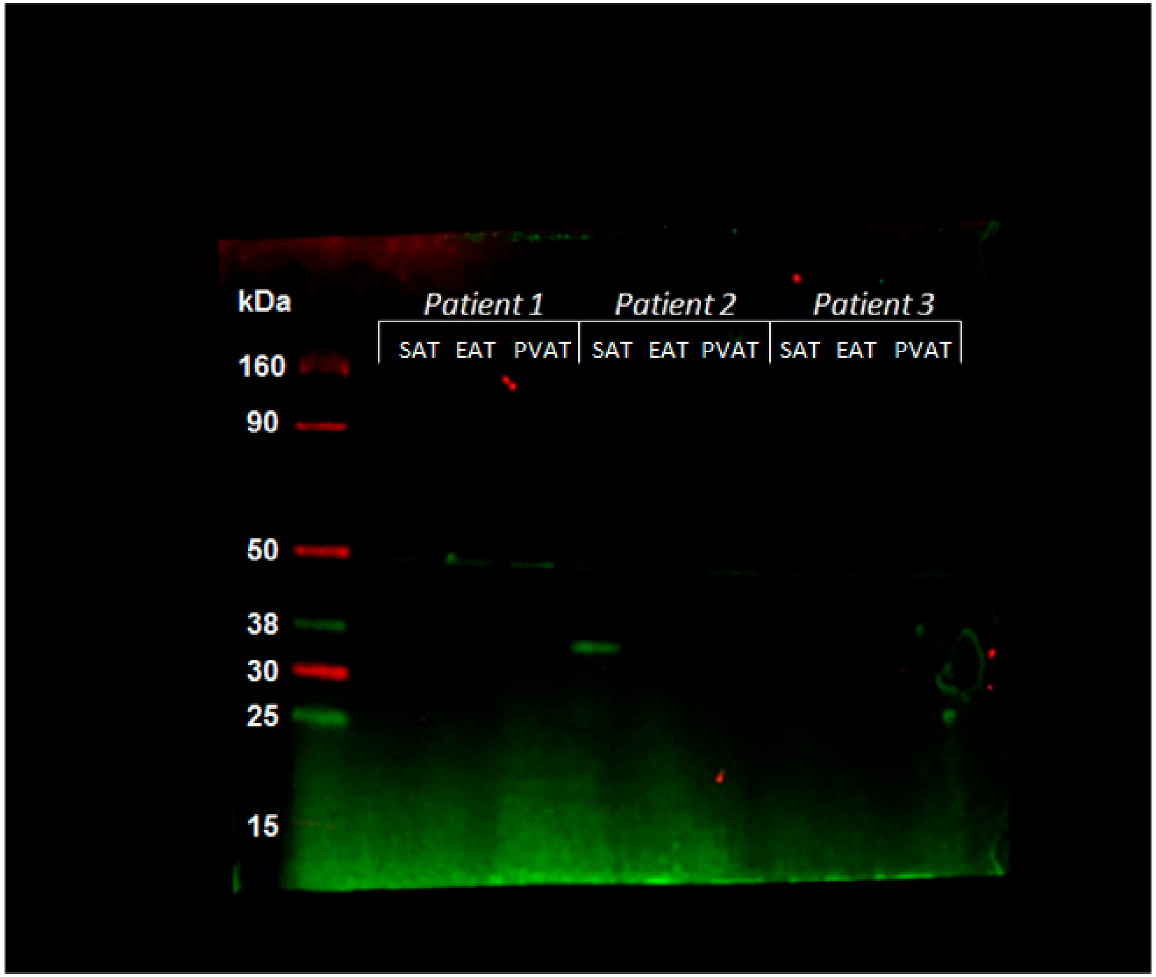

Supplementary Figure S7. Uncropped Western blot of DEGS1 expression in subcutaneous adipose tissue (SAT), epicardial adipose tissue (EAT), and perivascular adipose tissue (PVAT) in patients with coronary artery disease (CAD)

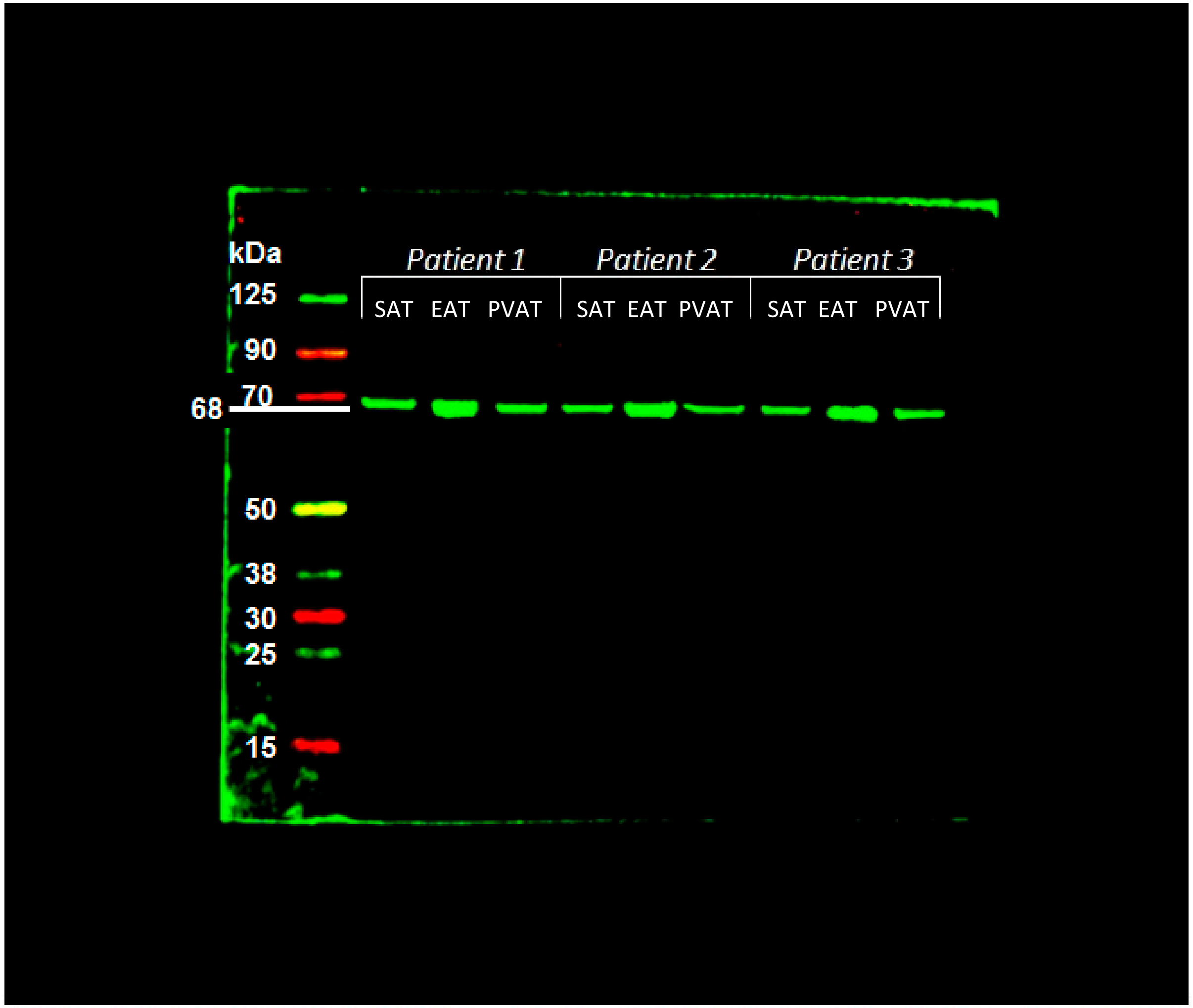

Supplementary Figure S8. Uncropped Western blot of DEGS1 expression in subcutaneous adipose tissue (SAT), epicardial adipose tissue (EAT), and perivascular adipose tissue (PVAT) in patients with valvular heart disease (VHD)

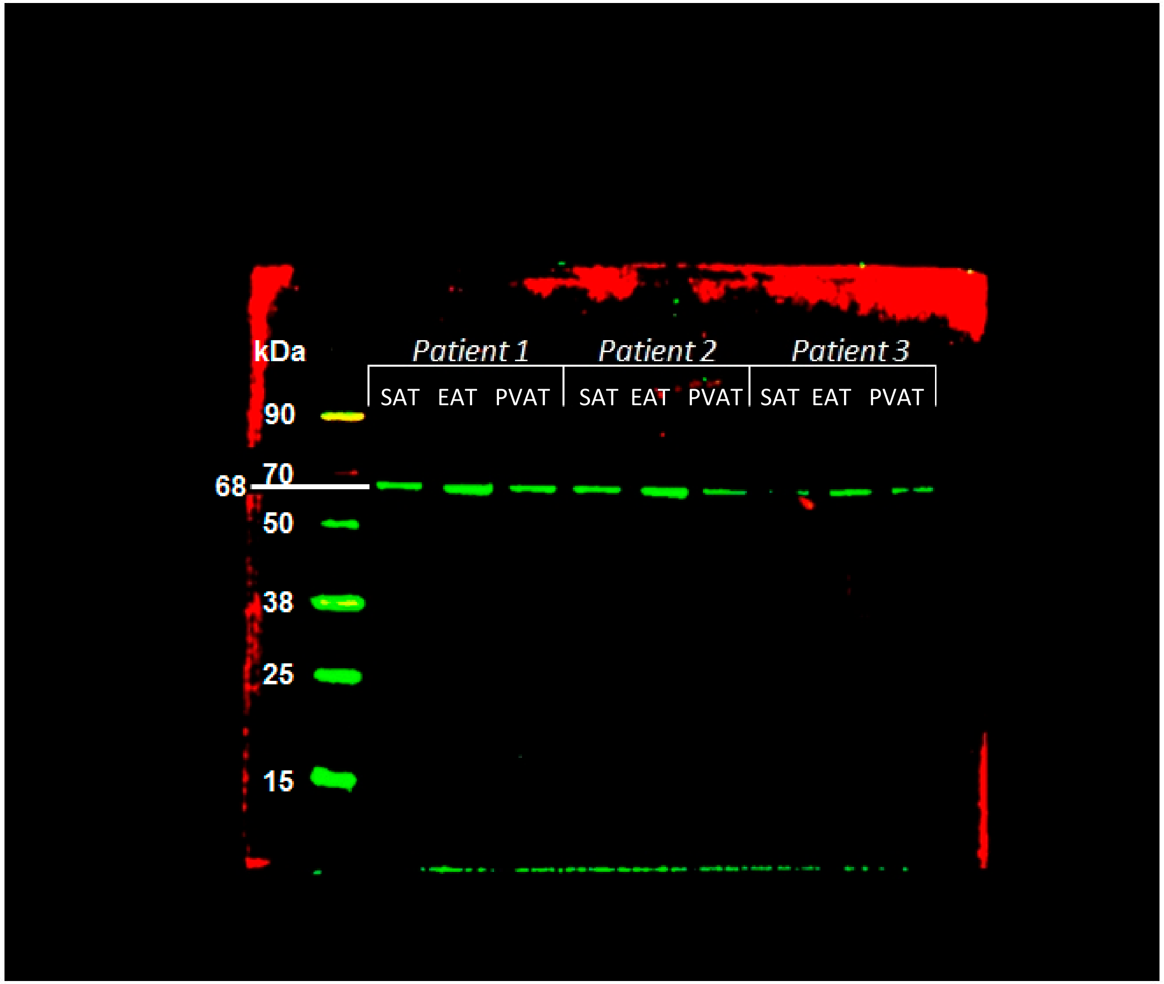

Supplementary Figure S9. Uncropped Western blot of ASMase expression in subcutaneous adipose tissue (SAT), epicardial adipose tissue (EAT), and perivascular adipose tissue (PVAT) in patients with coronary artery disease (CAD)

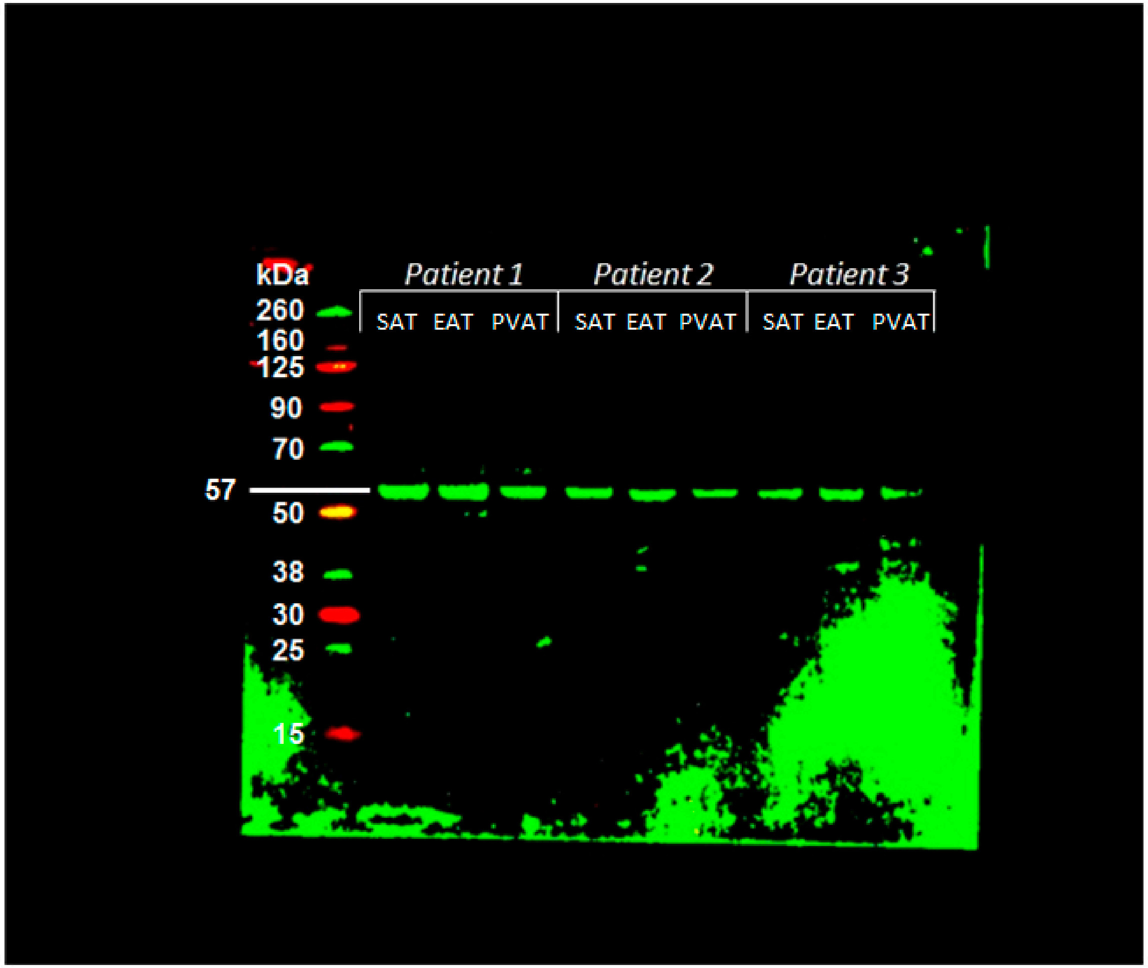

Supplementary Figure S10. Uncropped Western blot of ASMase expression in subcutaneous adipose tissue (SAT), epicardial adipose tissue (EAT), and perivascular adipose tissue (PVAT) in patients with valvular heart disease (VHD)

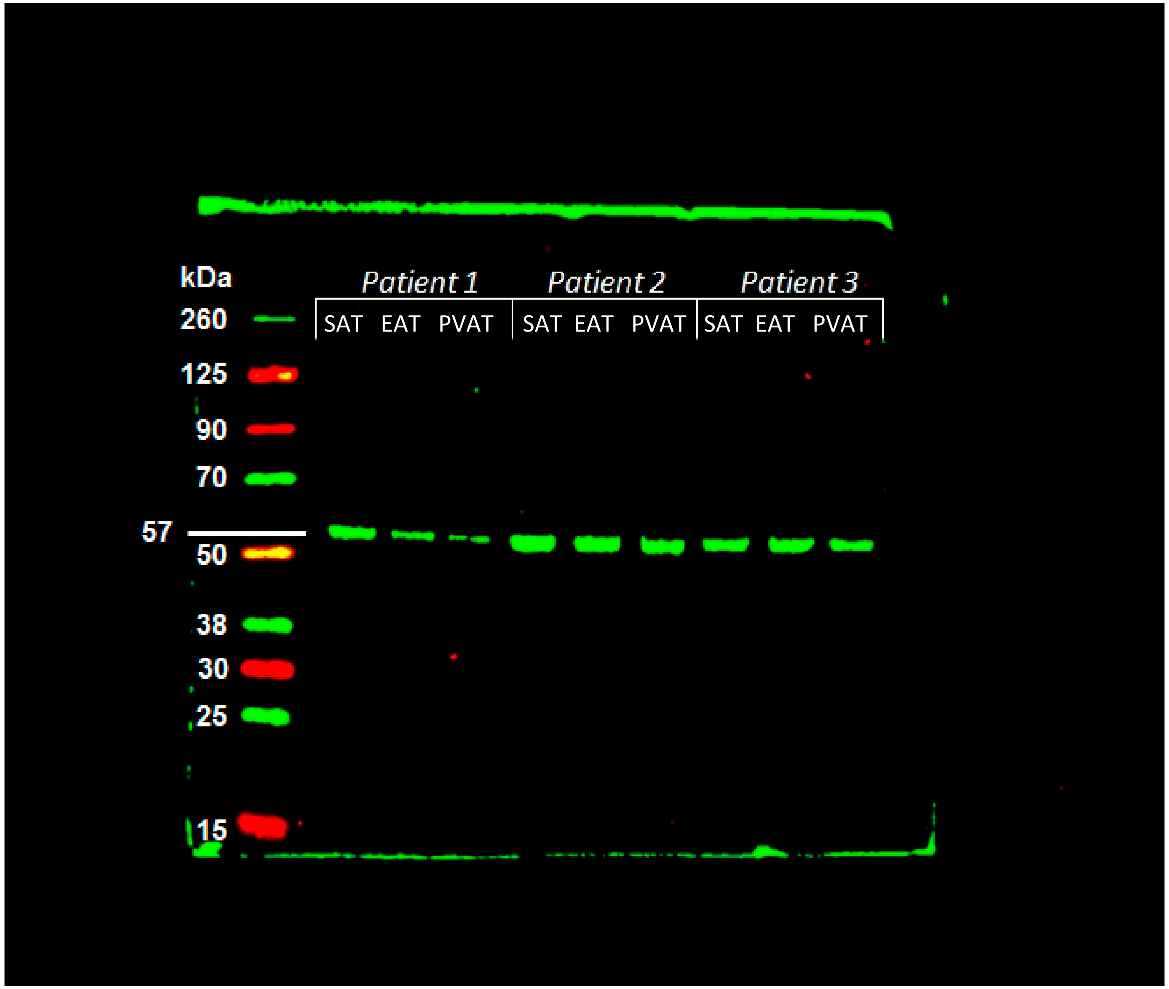

Supplementary Figure S11. Uncropped Western blot of NSMase2 expression in subcutaneous adipose tissue (SAT), epicardial adipose tissue (EAT), and perivascular adipose tissue (PVAT) in patients with coronary artery disease (CAD)

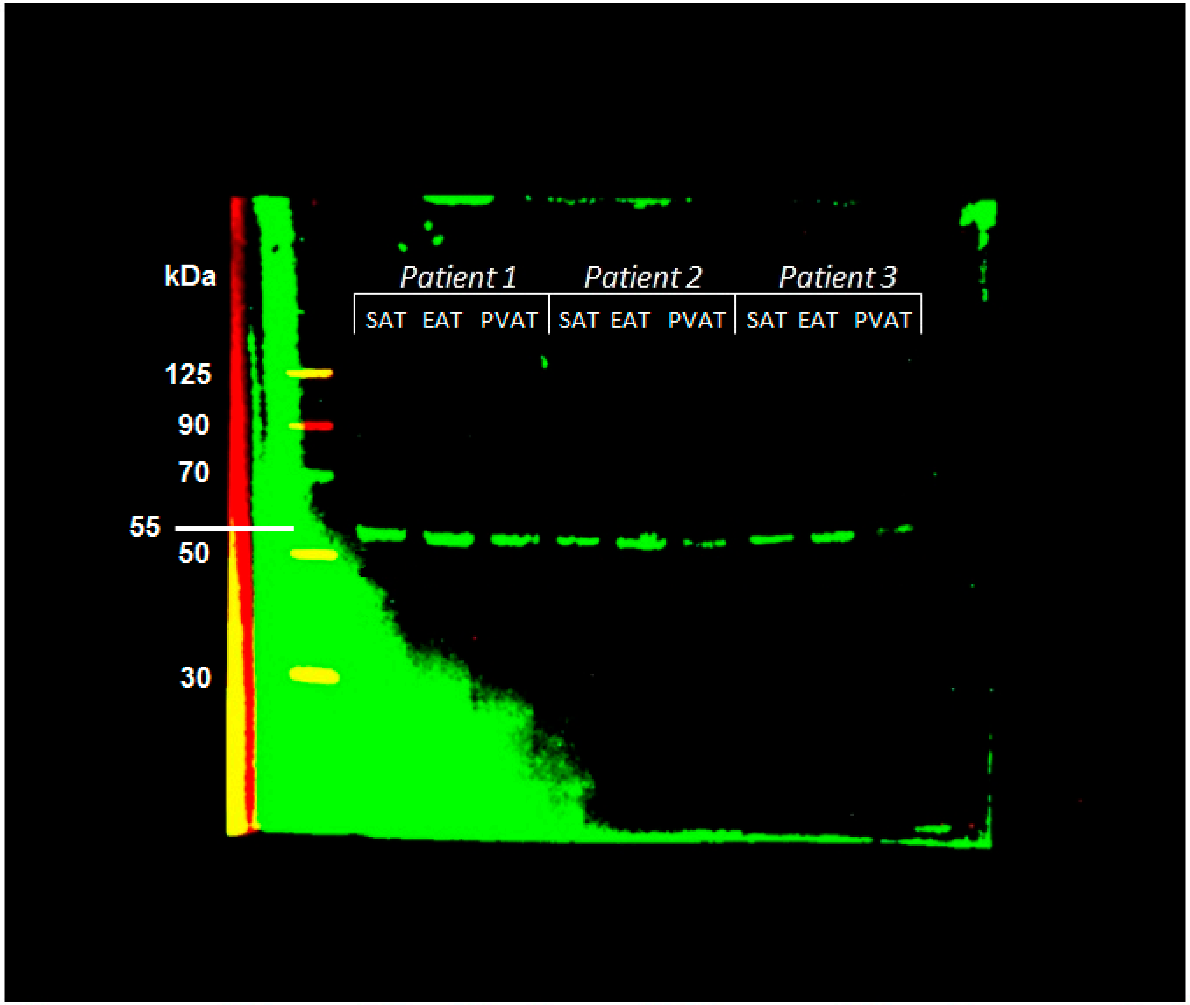

Supplementary Figure S12. Uncropped Western blot of NSMase2 expression in subcutaneous adipose tissue (SAT), epicardial adipose tissue (EAT), and perivascular adipose tissue (PVAT) in patients with valvular heart disease (VHD)

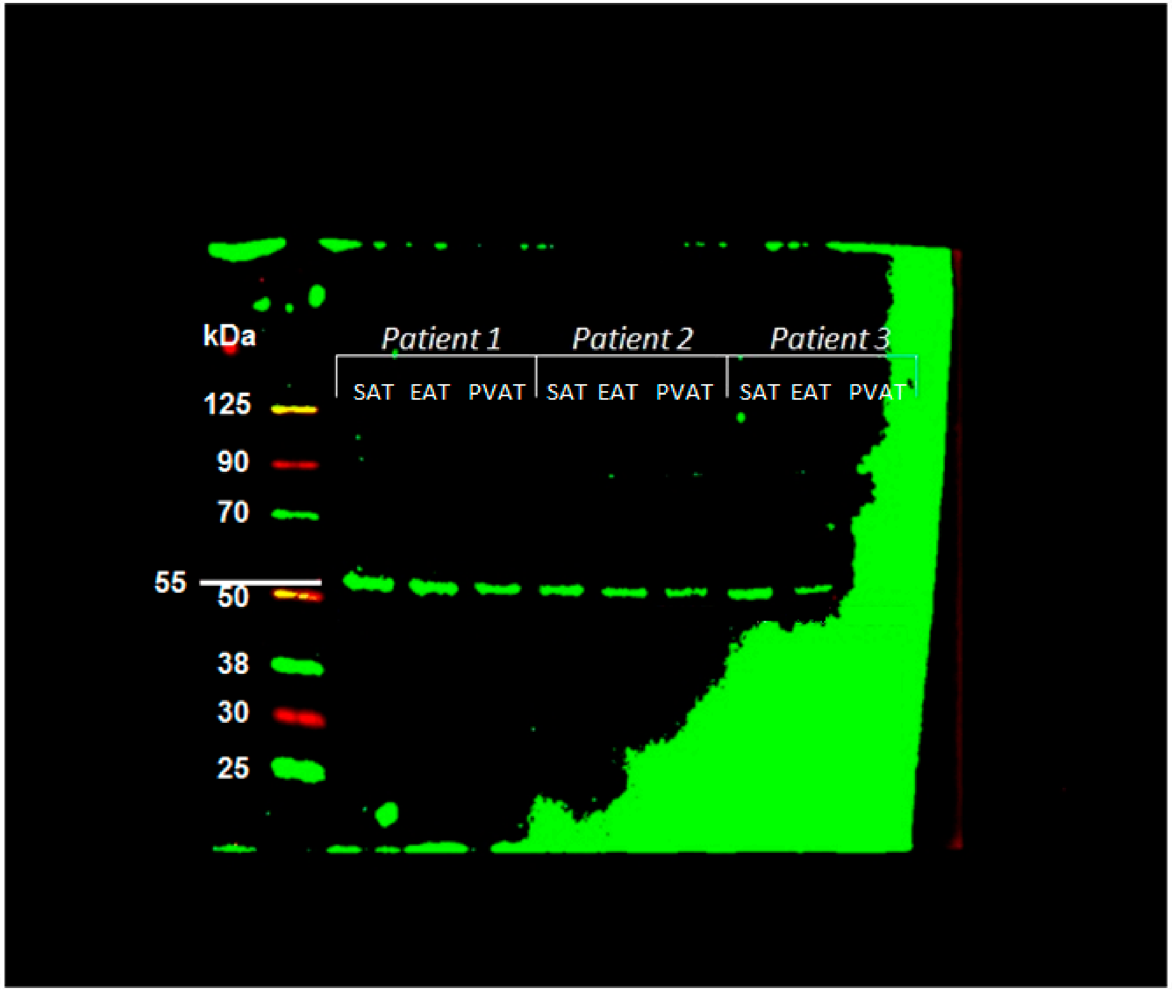

Supplementary Figure S13. Uncropped Western blot of ASAH1 expression in subcutaneous adipose tissue (SAT), epicardial adipose tissue (EAT), and perivascular adipose tissue (PVAT) in patients with coronary artery disease (CAD)

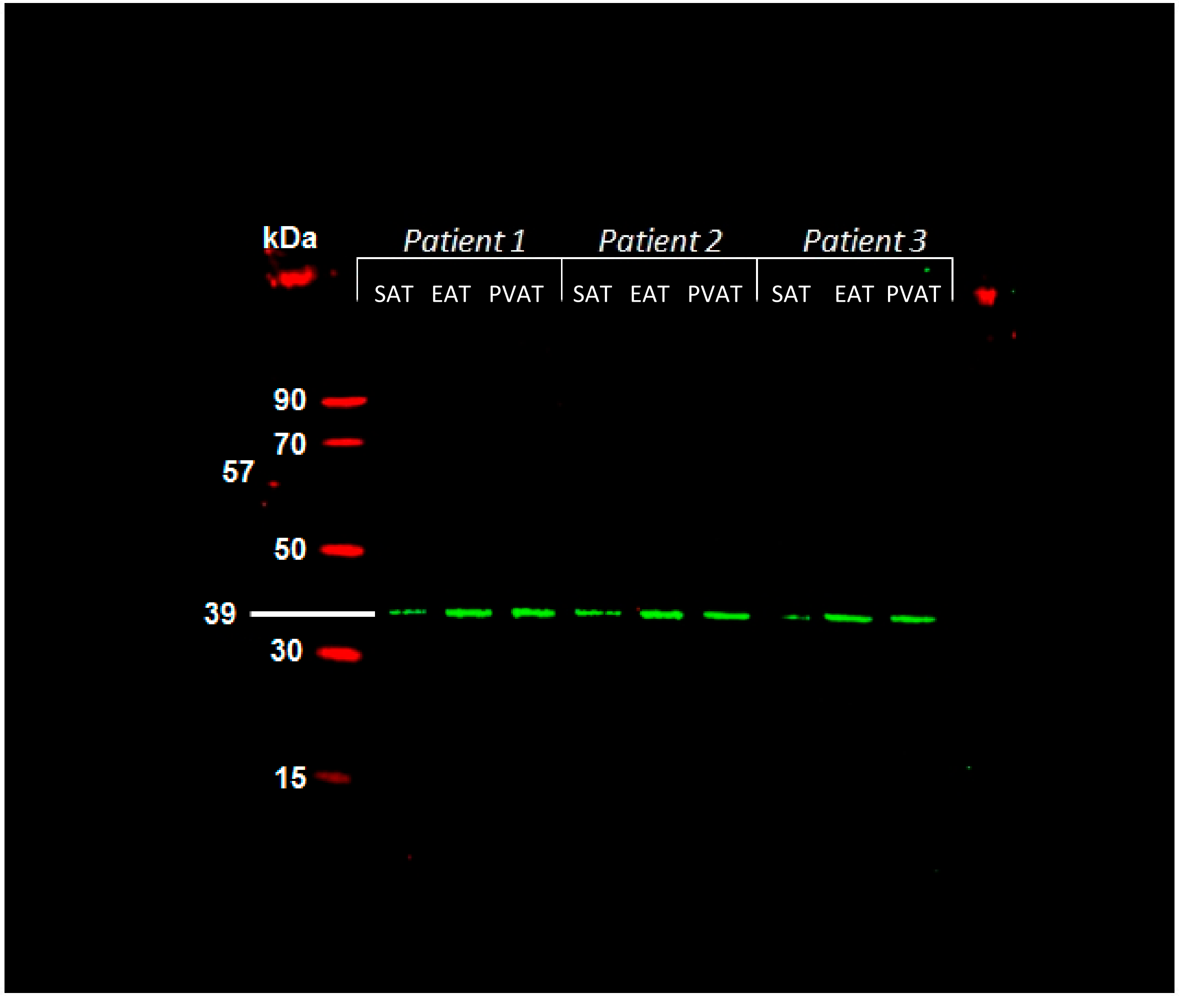

Supplementary Figure S14. Uncropped Western blot of ASAH1 expression in subcutaneous adipose tissue (SAT), epicardial adipose tissue (EAT), and perivascular adipose tissue (PVAT) in patients with valvular heart disease (VHD)

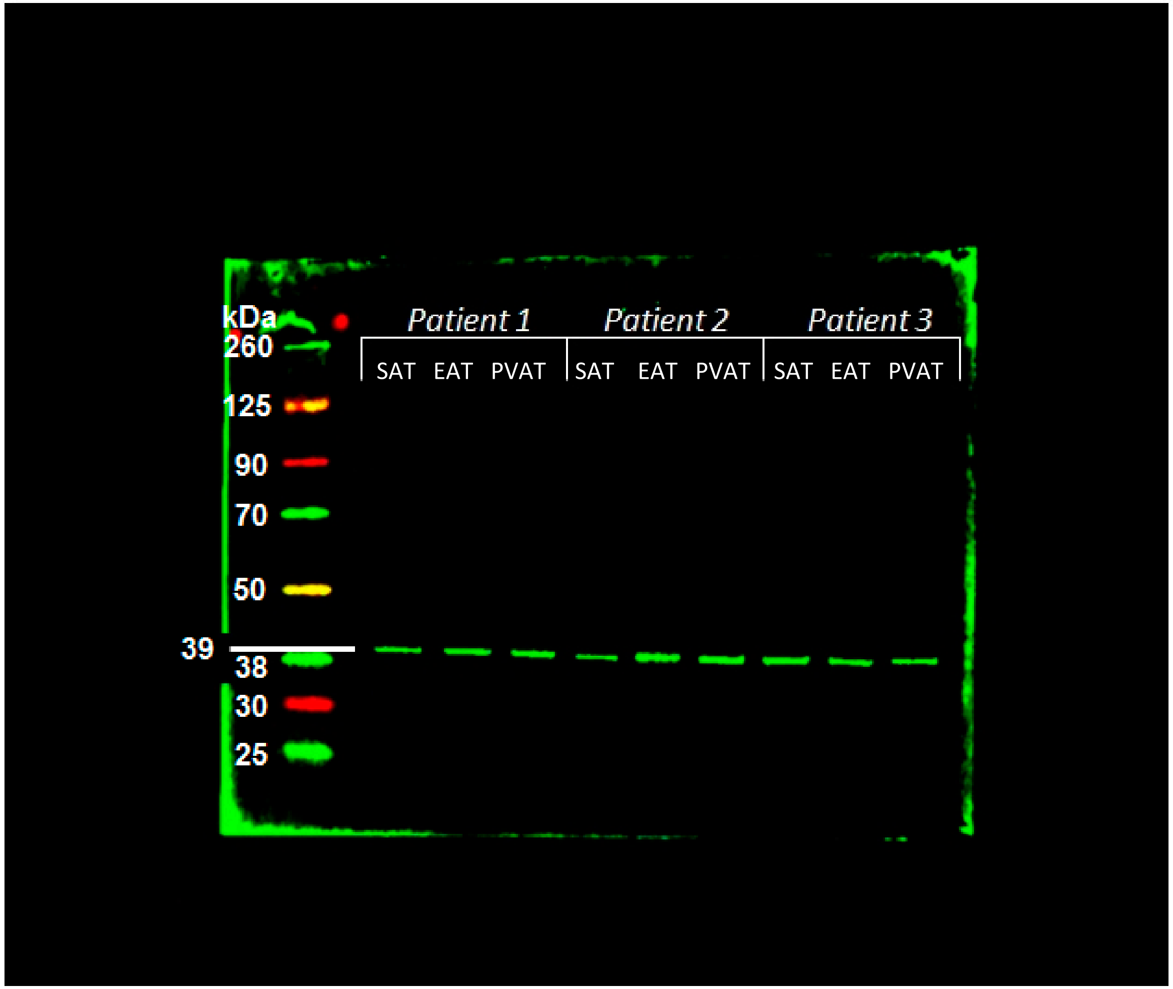

Supplementary Figure S15. Uncropped Western blot of SGMS1 expression in subcutaneous adipose tissue (SAT), epicardial adipose tissue (EAT), and perivascular adipose tissue (PVAT) in patients with coronary artery disease (CAD)

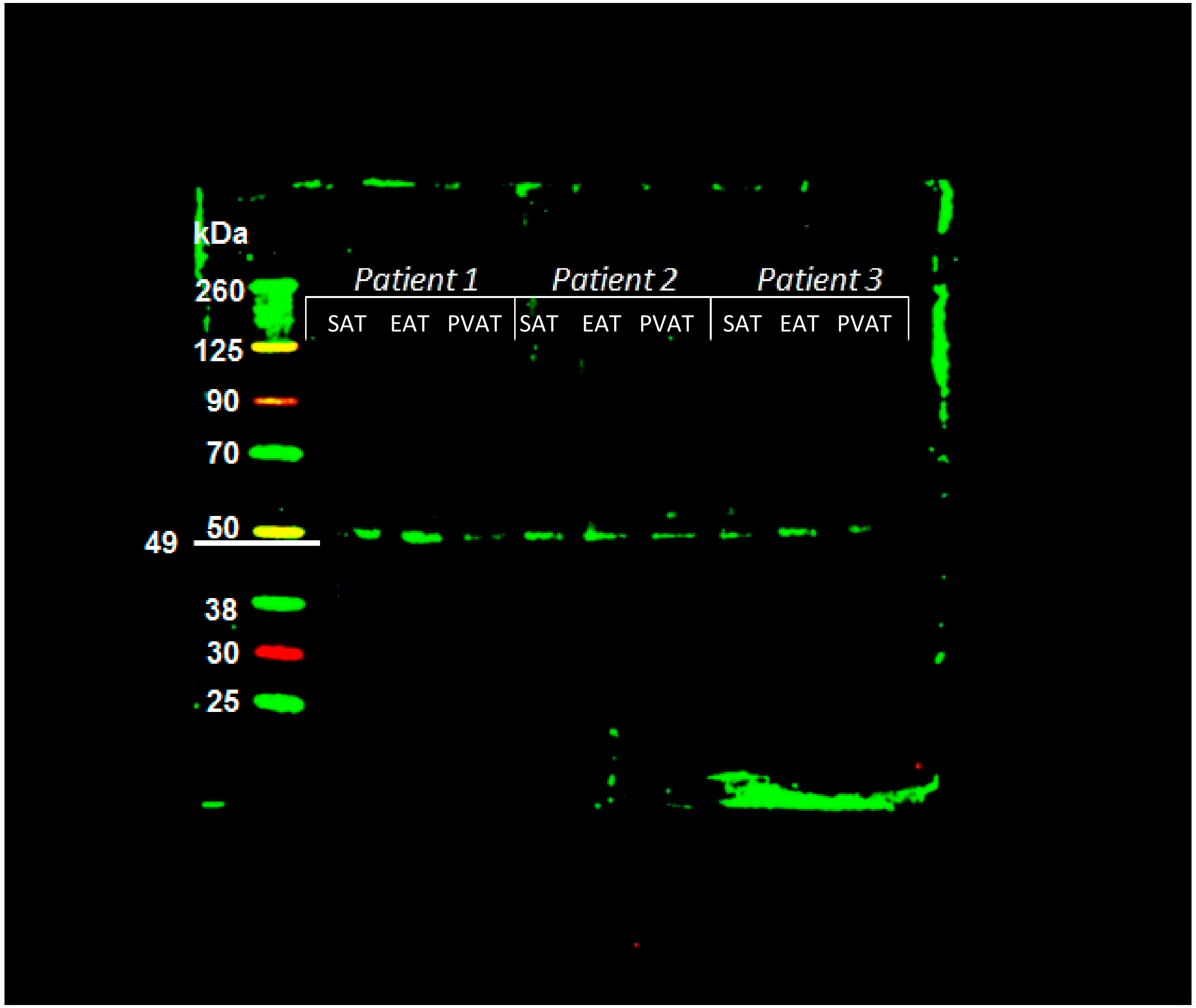

Supplementary Figure S16. Uncropped Western blot of SGMS1 expression in subcutaneous adipose tissue (SAT), epicardial adipose tissue (EAT), and perivascular adipose tissue (PVAT) in patients with valvular heart disease (VHD)

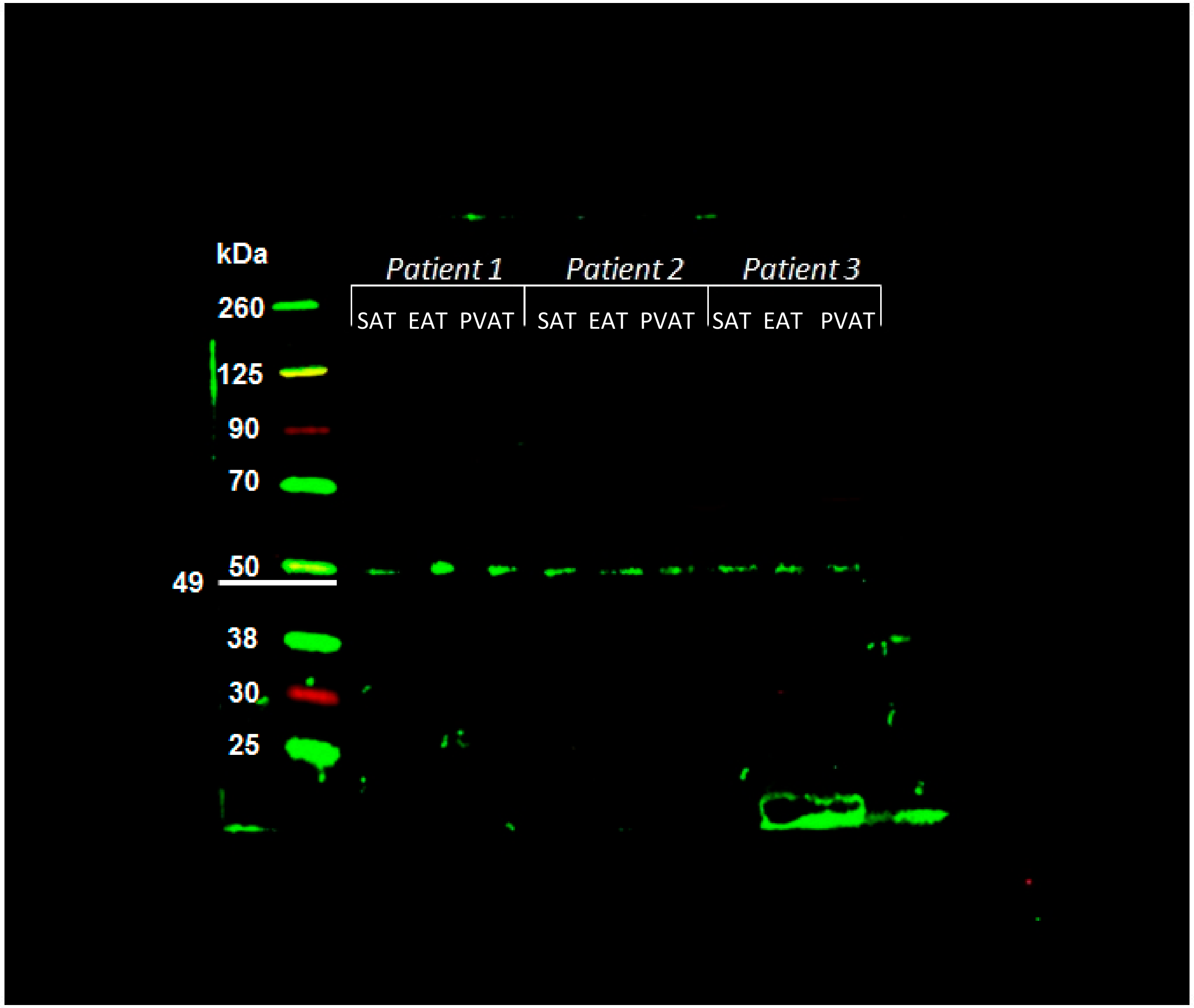

Supplementary Figure S17. Uncropped Western blot of SGMS2 expression in subcutaneous adipose tissue (SAT), epicardial adipose tissue (EAT), and perivascular adipose tissue (PVAT) in patients with coronary artery disease (CAD)

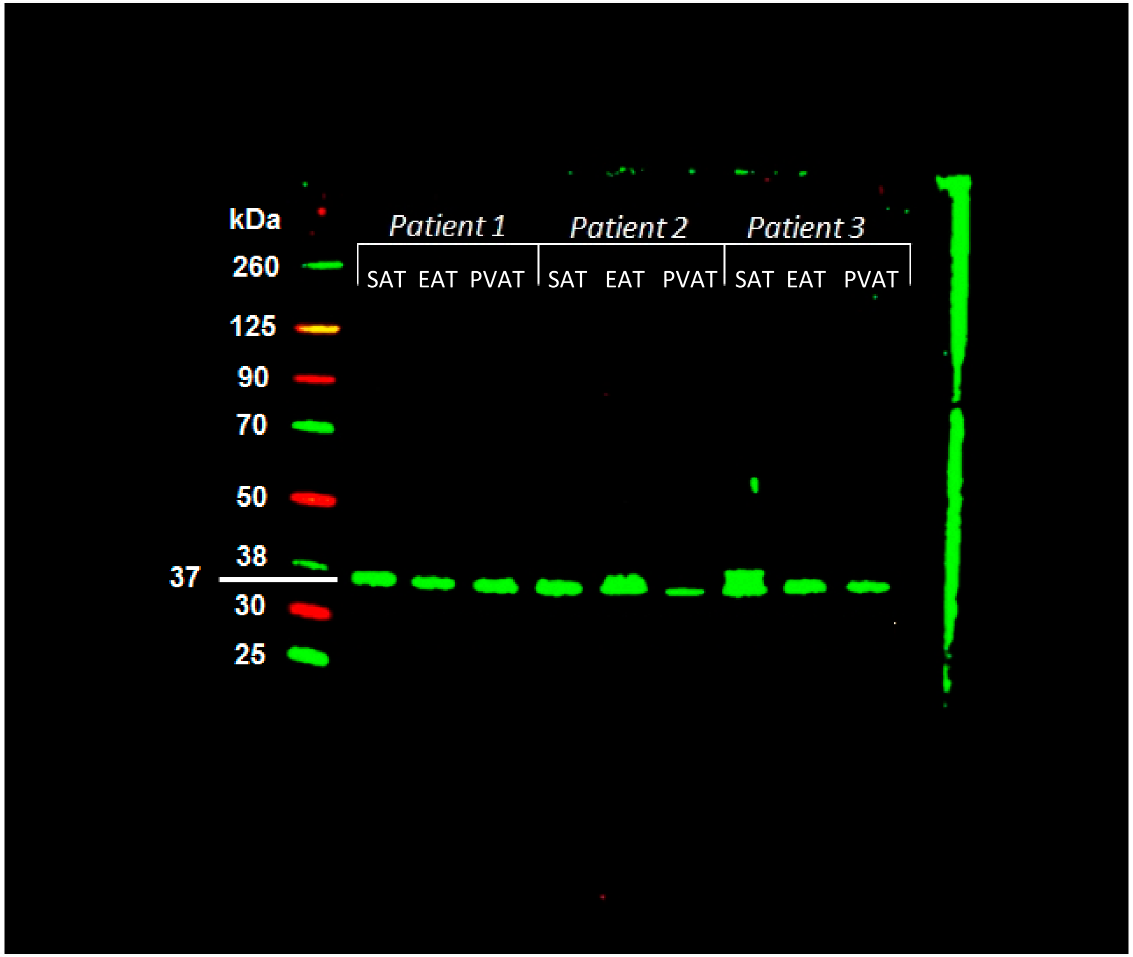

Supplementary Figure S18. Uncropped Western blot of SGMS2 expression in subcutaneous adipose tissue (SAT), epicardial adipose tissue (EAT), and perivascular adipose tissue (PVAT) in patients with valvular heart disease (VHD)

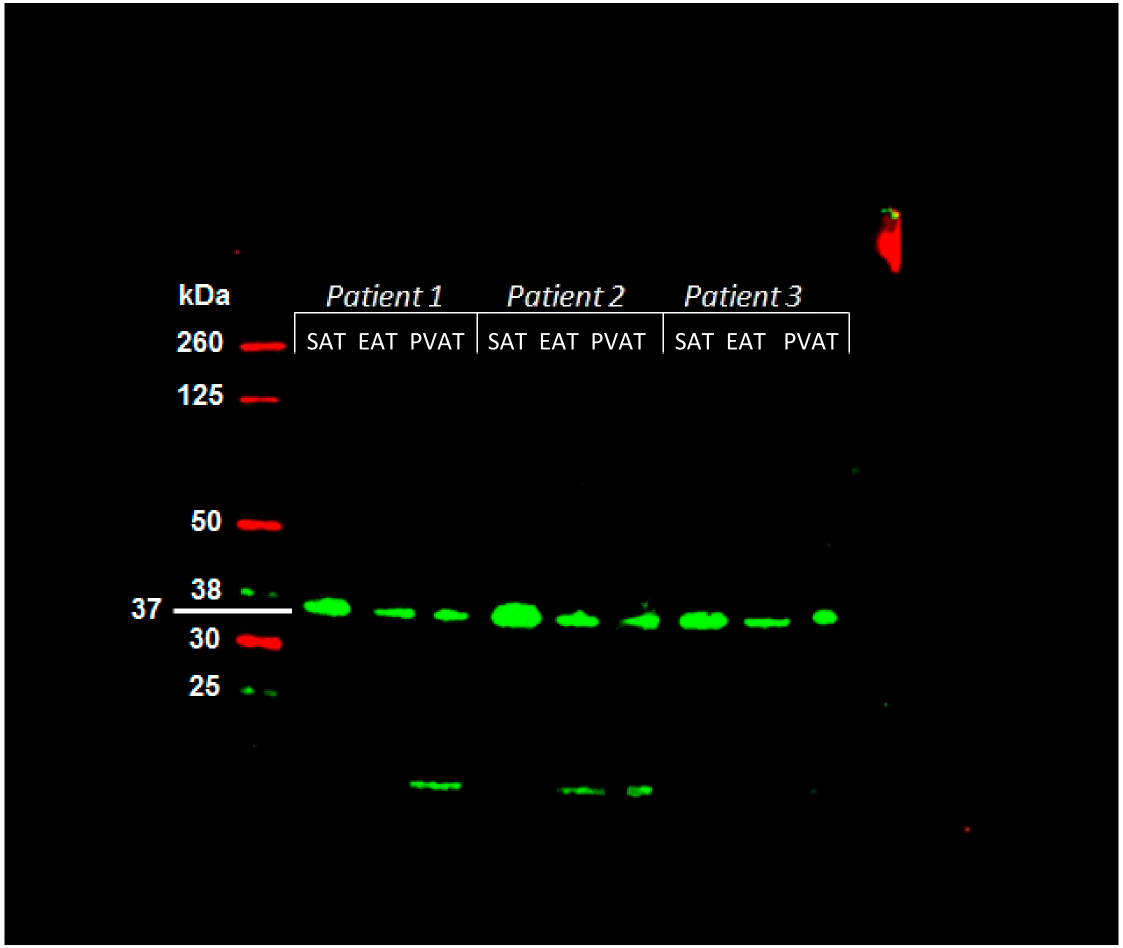

Supplement: Supplementary file 1 [file ijms-24-09494-s001.zip › ijms-2406882-supplementary.pdf]
